# The Validation and Implications of Using Whole Genome Sequencing as a Replacement for Traditional Serotyping for a National *Salmonella* Reference Laboratory

**DOI:** 10.3389/fmicb.2017.01044

**Published:** 2017-06-09

**Authors:** Chris A. Yachison, Catherine Yoshida, James Robertson, John H. E. Nash, Peter Kruczkiewicz, Eduardo N. Taboada, Matthew Walker, Aleisha Reimer, Sara Christianson, Anil Nichani, The PulseNet Canada Steering Committee, Ana Paccagnella, Celine Nadon

**Affiliations:** ^1^National Microbiology Laboratory, Public Health Agency of Canada, WinnipegMB, Canada; ^2^Department of Medical Microbiology, University of Manitoba, WinnipegMB, Canada; ^3^National Microbiology Laboratory, Public Health Agency of Canada, GuelphON, Canada; ^4^National Microbiology Laboratory, Public Health Agency of Canada, LethbridgeAB, Canada

**Keywords:** *Salmonella enterica*, whole genome sequencing, serotyping, serovars, surveillance, phenotype prediction

## Abstract

*Salmonella* serotyping remains the gold-standard tool for the classification of *Salmonella* isolates and forms the basis of Canada’s national surveillance program for this priority foodborne pathogen. Public health officials have been increasingly looking toward whole genome sequencing (WGS) to provide a large set of data from which all the relevant information about an isolate can be mined. However, rigorous validation and careful consideration of potential implications in the replacement of traditional surveillance methodologies with WGS data analysis tools is needed. Two *in silico* tools for *Salmonella* serotyping have been developed, the *Salmonella in silico* Typing Resource (SISTR) and SeqSero, while seven gene MLST for serovar prediction can be adapted for *in silico* analysis. All three analysis methods were assessed and compared to traditional serotyping techniques using a set of 813 verified clinical and laboratory isolates, including 492 Canadian clinical isolates and 321 isolates of human and non-human sources. Successful results were obtained for 94.8, 88.2, and 88.3% of the isolates tested using SISTR, SeqSero, and MLST, respectively, indicating all would be suitable for maintaining historical records, surveillance systems, and communication structures currently in place and the choice of the platform used will ultimately depend on the users need. Results also pointed to the need to reframe serotyping in the genomic era as a test to understand the genes that are carried by an isolate, one which is not necessarily congruent with what is antigenically expressed. The adoption of WGS for serotyping will provide the simultaneous collection of information that can be used by multiple programs within the current surveillance paradigm; however, this does not negate the importance of the various programs or the role of serotyping going forward.

## Introduction

*Salmonella enterica* is one of the most prevalent foodborne pathogens world-wide and a major causative agent of gastroenteritis in North America (Kim et al., 2006; Hendriksen et al., 2011; Parmley et al., 2013; Thomas et al., 2013; World Health Organization, 2015). Within Canada, *S.* *enterica* is estimated to be responsible for over 87,000 cases of domestically acquired foodborne illnesses each year (Thomas et al., 2013), leading to 11,600 hospitalizations and 238 deaths (Thomas et al., 2015). In Canada, the surveillance of this priority pathogen is a multifaceted endeavor, encompassing multiple surveillance programs that monitor *Salmonella* along the farm to fork to human clinical disease spectrum (Parmley et al., 2013). While the individual mandates of the various surveillance systems differ (Swaminathan et al., 2006; Government of Canada, 2014), all heavily rely on the classification of isolates into serovars by the globally recognized serotyping system (Parmley et al., 2013); the White-Kauffmann-Le Minor (WKL) scheme (Grimont and Weill, 2007).

The *Salmonella* serotyping system classifies isolates on the basis of the immunological reactions to cell surface antigens, specifically the somatic O and the two variably expressed flagellar H antigens, denoted H1 and H2 (Kim et al., 2006; Wattiau et al., 2011; Zhang et al., 2015; Yoshida et al., 2016b). The most recent edition of the WKL scheme has identified over 2,500 serovars belonging to the five subspecies of *S. enterica* (Grimont and Weill, 2007). However, it is important to note that the majority of human clinical disease is the result of a select few important human pathogenic serovars (Hendriksen et al., 2011). Traditionally, serotyping is performed through the phenotypic characterization of the O and H antigens via the slide agglutination test, in which the clumping of cells is observed in response to specific antisera. Although this technique is widely used (Wattiau et al., 2011), it can be time consuming and laborious (Kim et al., 2006; Wattiau et al., 2011; Zhang et al., 2015), and still leave five to eight percent of all *Salmonella* isolates untypeable (Kim et al., 2006).

Increasingly, the global food-safety and public health communities are looking toward the ‘omics’ technologies to provide a rapid, cost effective, high throughput, and expansive set of data, from which all the relevant information about an isolate or organism can be mined (Bergholz et al., 2014; Ronholm et al., 2016). The technological advancements of the whole genome sequencing (WGS) platforms, such as the Illumina MiSeq, and the improved bioinformatic analyses are revolutionizing surveillance programs. WGS promises to not only provide the best dataset available to determine pathogen relatedness, fulfilling a key mandate of programs such as PulseNet Canada, but WGS data could also be used to derive information about other important characteristics, such as serotype, using *in silico* workflows (Gilmour et al., 2013; Ronholm et al., 2016).

In recent years, multiple applications for the *in silico* determination of *Salmonella* serovars from WGS data have been developed (Achtman et al., 2012; Zhang et al., 2015; Yoshida et al., 2016b). Achtman et al. (2012) suggested that the serovar of an isolate could be inferred from multi-locus sequence typing (MLST) (Achtman et al., 2012). MLST assigns unique alleles to different sequences across 400–500bp fragments of specified housekeeping genes (loci). The alleles assigned across all the loci are used to define the individual sequence types (ST), converting a large amount of data into an easily defined single number (Maiden et al., 1998). In the MLST serotyping scheme developed for *Salmonella* seven housekeeping gene fragments are typed and the individual ST or their larger clonal complexes are then linked to *Salmonella* serovars. While MLST is not strictly an *in silico* analysis, the MLST data can be mined directly from WGS data using bioinformatic pipelines (Achtman et al., 2012; Ashton et al., 2016). Applications such as SeqSero^1^ and the *Salmonella in silico* Typing Resource (SISTR^2^) have utilized an approach in which the gene sequences encoding the individual somatic and flagellar antigens are used to determine an isolate’s serovar. SeqSero extracts the relevant genomic regions, specifically the *rfb* region for somatic antigen determination and the *fliC* and *fljB* genes for H1 and H2 antigen determination, from the genome assemblies and aligns these regions to curated databases (Zhang et al., 2015) using BLAST (Basic Local Alignment Search Tool) (Camacho et al., 2009). The overall antigenic formula is then looked up in the WKL, much like traditional serotyping (Zhang et al., 2015). The SISTR platform looks at the highly variable *wzx* and *wzy* genes of the *rfb* region to determine the somatic antigen, and the *fliC* and *fljB* genes to determine the H1 and H2 antigens, respectively. The predicted antigenic formula is queried against the WKL serovar database to provide a serovar name. Unlike flagellar antigens, the somatic O antigens are the result of complex molecular pathways and at present there is not enough known about the biology of the organism to make predictions of the individual O antigens expressed, but using the information present in the *wzx*/*wzy* genes it is possible to assign isolates to a serogroup. Additional testing may be required to resolve the ambiguous serovar designations as a result of the serogroup vs individual antigen determination. To resolve these ambiguities SISTR uses a novel 330 locus core genome MLST (cgMLST) analysis to provide a phylogenetic context; where the predominant serovar within the cgMLST cluster is used to choose the most likely serovar from the list of potential serovars. Together these two methodologies are used to provide an overall SISTR serovar prediction with improved concordance with traditional methods (Yoshida et al., 2016b).

Before WGS data can be used for the serotyping of *Salmonella*, there needs to be rigorous validation of any proposed method and careful implementation plans must be prepared to ensure the data is appropriate to replace the traditional serotyping method so there is minimal disruption to current surveillance programs. Validation of the *in silico* serotyping tools needs to be performed using a heavily curated panel of isolates with reliable identifications to ensure that the produced results are accurate (Gilmour et al., 2013). The objective of this project was to: validate and compare *in silico* serotyping methodologies, MLST, SeqSero, and SISTR; assess the impact of *in silico* serotyping on existing surveillance systems; and develop the considerations that can guide the future implementation of *in silico* serotyping for national surveillance in Canada and elsewhere.

## Material and Methods

### Study Design

A total of 813 *Salmonella enterica* isolates were selected for this study to assess the performance of the three *in silico* serotyping methods, SISTR, SeqSero, and MLST in comparison to phenotypic serotyping. Results from the validation were used to identify the implications of using an *in silico* serotyping tool and develop considerations for the replacement of traditional serotyping with said tool.

### Study Isolates and Traditional Serotyping

All isolates used in this study were previously serotyped using traditional methods as follows. Isolates were grown overnight at 37°C on Luria-Bertani agar (BD Canada, Mississauga, ON, Canada) and the antigenic formula of each strain was determined using standard methods (Shipp and Rowe, 1980; Ewing, 1986), with the serovar assigned according to the WKL scheme in an OIE/ISO accredited laboratory (Grimont and Weill, 2007). Isolates were all phenotypically serotyped in the Reference Laboratory at the National Microbiology Laboratory (NML).

The 813 isolates used in this study could be split into two groups on the basis of their rationale for inclusion in the study. Group one consisted of 492 isolates from multiple target serovars that were chosen for their clinical relevance and importance in diagnostic and reference laboratories serving surveillance functions in Canada. Four-hundred of the 492 isolates from this group were split evenly among the top twenty serovars reported to NESP in 2012. Together these serovars represent about 85% of all reported cases of human salmonellosis in Canada (Government of Canada, 2014), and include serovars: Enteritidis, Heidelberg, Typhimurium, ssp I 4,[5],12,:i:-, Thompson, Infantis, Newport, Typhi, ssp I 4,[5],12:b:-, Braenderup, Saintpaul, Javiana, Paratyphi A, Hadar, Agona, Paratyphi B var. Java, Stanley, Oranienburg, Muenchen, and Montevideo. The remaining 92 isolates from this group represented a collection of clinically relevant but infrequently encountered serovars in Canada. These 92 isolates included: serovars of increased clinical importance, either due to increased association with invasive or travel related infections (specifically serovars Dublin, Cerro, Schwarzengrund, Sandiego, Panama, and Corvallis); serovars deemed difficult to differentiate by traditional serotyping (specifically serovars Senftenberg and Kouka; Carrau and Madelia; Lattenkamp; and Paratyphi B); serovars from non-subspecies I (specifically from subspecies II, IIIa, IIIb, and IV); and isolates left untypeable by traditional serotyping. All isolates collected as part of this group were randomly selected from the total population of *Salmonella* isolates from their respective serovars that were submitted to the NML in Winnipeg between the years 2009–2013. Group two consisted of 321 isolates chosen to represent the most globally prevalent *Salmonella* serovars from both human and non-human sources. The isolates from this group were previously used in a validation study evaluating other molecular typing methods and were collected from human, animal, and environmental sources (Yoshida et al., 2016a). One hundred and fifty-two isolates in this grouping were from the target serovars outlined above, while the other 169 isolates in this group were from a collection of other serovars, meant to provide coverage for the majority of antigenic determinants currently described.

### Genome Sequencing and Assembly

Genomic DNA was extracted from group one isolates using overnight cultures grown in LB-Lennox 0.5% NaCl broth via the Qiagen DNeasy 96 Blood and Tissue Kit (Qiagen Ltd., Mississauga, ON, Canada) or from a nutrient agar plate using the Epicenter Metagenomics DNA isolation kit for water (Mandel Scientific Company Inc., Guelph, ON, Canada). For isolates from group two, genomic DNA was extracted from isolated colonies on overnight Luria_Bertani agar plates using the KingFisher Cell and Tissue DNA Kit (VWR, Mississauga, ON, Canada) on the KingFisher Flex (VWR) or using the EZ1 DNA tissue kit and BioRobot (Qiagen). Manufacturer’s instructions were followed with the addition of 100 g of lysozyme (Sigma-Aldrich Canada Ltd., Oakville, ON, Canada; 10 mg/ml) in the cell lysis incubation stage for isolates from group two.

Recovered DNA for all isolates in the study was quantified with a Qubit DNA quantification system (Invitrogen Canada Inc., Burlington, ON, Canada) and diluted down to a genomic DNA concentration of 0.2 ng/μl. Sample libraries for all isolates were prepared using the MiSeq Nextera XT library preparation kit (Illumina, Inc., San Diego, CA, United States). Libraries were size selected for a minimum insert size of 500bp using the BluePippin (Sage Science, Beverly, MA, United States). Paired end sequencing was performed on the Illumina MiSeq with the MiSeq Reagent Kit v3 600 cycles (2 × 300bp forward and reverse) to achieve an average estimated genome coverage greater than 30× for all isolates. Raw sequencing read data was uploaded to NCBI for all isolates. Group one isolates have been uploaded under BioProject PRJNA353625, while isolates from group two have been uploaded under BioProject PRJNA354244.

The paired end reads were first merged using FLASH (version 1.2.9^3^) (Magoc and Salzberg, 2011) and then *de novo* assembled into contigs using SPAdes (version 3.5.0^4^) (Bankevich et al., 2012). SPAdes was run using the careful option to correct assembly errors and the resulting FASTA files were used in downstream analysis.

### Interpretation and Scoring of Results

All isolates were uploaded to the SISTR website^5^ (Yoshida et al., 2016b) and SeqSero website^6^ (Zhang et al., 2015) for serovar prediction. An *in silico* 7-gene MLST ST was generated using the SISTR platform and ST data from the platform was compared to the University of Warwick *Salmonella enterica* MLST database^7^ to generate the MLST serovar prediction (Achtman et al., 2012). A comparison of results to traditional serotyping was done using the interpretation criteria as described below. The results were categorized into full, inconclusive, incongruent or incorrect matches. Matches were considered “full” when the overall serovar prediction was concordant with the reported serovar by traditional typing. Matches were considered “inconclusive” when the overall serovar prediction was ambiguous (multiple serovars indicated), or of partial prediction (information was missing in overall prediction, but the individual parts provided were correct). Matches were considered “incongruent” when the overall serovar prediction was incongruent with the reported phenotypic serovar due to the carriage of antigenic determinants (either phase two flagellar or somatic antigen genes) that were not expressed phenotypically. Matches were considered “incorrect” when the overall serovar prediction was incorrect with respect to the reported serovar by traditional serotyping. Successful predictions were calculated based on the proportion of results that were categorized as full, inconclusive, or incongruent matches, indicating a positive test result in relation to traditional serotyping.

A Fisher’s exact test was used to evaluate the statistical significance between the successful predictions from each platform using a 2-by-2 contingency table via Graphpad Quickcalcs^8^. A *P*-value of less than or equal to 0.05 was considered statistically significant.

The test sensitivity and specificity for the prediction of Enteritidis and Typhimurium was also assessed for each platform in comparison to standard serotyping through a 2-by-2 table analysis (Mackinnon, 2000), following the removal of any incongruent results from the analyses. Incongruent results were removed from the analysis due to their inherent incompatibility between the phenotypically and genetically derived test results which could artificially reduce the sensitivity and specificity measured. Test sensitivity was defined as TP/(TP+FN) and test specificity was defined as TN/(TN+FP), where TP = true positives, FN = false negative, TN = true negatives, and FP = false positives.

## Results

The performance of the three *in silico* methods for *Salmonella* serovar prediction was assessed using a panel of 813 isolates, including 492 Canadian clinical isolates and 321 isolates of human and non-human sources. The three methods, SISTR, SeqSero, and MLST provided successful results for 94.8, 88.2, and 88.3% of the 813 isolates tested, respectively; where successful results were considered when the result did not include incorrect information. These successful identifications were further broken down into full matches, inconclusive matches, and incongruent matches. Full matches were recorded when there was an identical serovar match between traditional serotyping and the *in silico* method under study. We recorded full matches in 89.7, 54.1, and 77.9% of the isolates tested using SISTR, SeqSero, and MLST, respectively. Inconclusive matches were recorded when the information provided by the *in silico* typing method was insufficient and would require further laboratory analysis to determine or narrow down the correct serovar. Inconclusive matches were reported with SISTR, SeqSero, and MLST for 1.1, 30.0, and 6.4%, respectively, of the isolates tested. Incongruent matches were when the phenotypic serovar prediction was incongruent with the *in silico* prediction due to the carriage of unexpressed antigenic determinants. We noted incongruent matches in 4.1% of the isolates tested regardless of testing method. Lastly, incorrect results were reported in 5.2, 11.8, and 11.7% of the isolates tested using SISTR, SeqSero, and MLST, respectively. A summary of these results is presented in **Table 1**. A breakdown of the results for individual serovars within the target group is included in Supplementary Table 1. The number of successful results reported by SISTR was deemed to be significantly greater than the number of successful results reported by either SeqSero or MLST (one-tailed *p*-values of less than 0.001). However, the observed differences in number of successful results recorded by SeqSero and MLST was not deemed to be statistically significant.

**Table 1:** Performance of the three *in silico* methods for *Salmonella* serovar prediction, SISTR, SeqSero, and MLST, compared to traditional serotyping for 813 *Salmonella enterica* isolates.

Platform	Successful results			Total successful results	Incorrect results	Total tested
	**Full Match**	**Inconclusive Match**	**Incongruent Match**			
SISTR	729 (89.7%)	9 (1.1%)	33 (4.1%)	771^∗^ (94.8%)	42 (5.2%)	813
SeqSero	440 (54.1%)	244 (30.0%)	33 (4.1%)	717 (88.2%)	96 (11.8%)	813
MLST	633 (77.9%)	52 (6.4%)	33 (4.1%)	718 (88.3%)	95 (11.7%)	813

*^∗^Statistically significant improvement compared to other *in silico* platforms, one-tailed *p*-values ≤ 0.001.*

All rough isolates were naturally considered to be incongruent matches, and a complete serovar call was generated for 96, 54, and 85% of the rough isolates tested by SISTR, SeqSero, and MLST analysis methods, respectively. While partial results were generated for all isolates tested using SISTR and SeqSero and for 85% of the isolates tested using MLST analysis. Predictions across the platforms were consistent with each other and any reported H antigens, except for one MLST predictions which were inconsistent with the reported H antigens, and one isolate whose H2 antigen prediction differed between SISTR and SeqSero, but could not be typed via traditional serotyping. No single genetic change or phylogenetic signal was detected amongst the 26 rough isolates tested as part of our study.

Test sensitivity and specificity was calculated for each *in silico* serotyping method for serovars Enteritidis and Typhimurium due to their increased global prevalence and importance. Test sensitivity for serovar Enteritidis was 95.2, 97.1, and 87.0% for the SISTR, SeqSero, and MLST methods, respectively; while test specificity for serovar Enteritidis was 99.7, 98.9, and 99.7%. SISTR, SeqSero, and MLST displayed test sensitivities of 92.9, 100, and 65% and test specificities of 100, 98.3, and 100% for serovar Typhimurium, respectively. A summary of the test specificity and test sensitivity results for these two serovars across the analysis methods is displayed in **Table 2**.

**Table 2:** Sensitivity and specificity of prediction of *Salmonella* serovars Enteritidis and Typhimurium using three *in silico* methods for *Salmonella* serovar determination, SISTR, SeqSero, and MLST.

	Method	TP^1^	TN^2^	FP^3^	FN^4^	Total tested	Sensitivity	Specificity
*Salmonella* serovar Enteritidis	SISTR	40	736	2	2	780	95.2	99.7
	SeqSero	34	737	1	8	780	97.1	98.9
	MLST	40	732	6	2	780	87.0	99.7
*Salmonella* serovar Typhimurium	SISTR	39	738	3	0	780	92.9	100
	SeqSero	26	741	0	13	780	100	98.3
	MLST	39	720	21	0	780	65.0	100

*^1^True positives; ^2^True negatives; ^3^False positives; ^4^False negatives.*

## Discussion

Two platforms have been developed for *in silico Salmonella* serovar prediction, SISTR (Yoshida et al., 2016b), and SeqSero (Zhang et al., 2015), while 7-gene MLST analysis (Achtman et al., 2012) can be adapted as an *in silico* serovar test (Ashton et al., 2016) and all were assessed in the current study. We report that successful results were obtained for 94.8, 88.2, and 88.3% of the 813 *Salmonella* isolates tested using SISTR, SeqSero, and MLST, respectively (**Table 1**). These data are largely consistent with the previously reported success rate of each individual platform (Zhang et al., 2015; Ashton et al., 2016; Yoshida et al., 2016b). In our study SISTR significantly outperformed both SeqSero and MLST analysis for the *in silico* serotyping of isolates. Previous global surveys of quality assurance testing for traditional *Salmonella* serotyping methods have found participating laboratories worldwide were able to correctly identify an average of 82% of stains tested (Hendriksen et al., 2009), and this shows the overall suitability of replacing phenotypic serotyping with an *in silico* analysis tool. The test sensitivity and specificity of the platforms for serovars Enteritidis and Typhimurium was also assessed. The test sensitivity values ranged from a low of 65% for serovar Typhimurium using the MLST analysis platform to a high 100% for the same serovar using the SeqSero platform, with the rest of the values falling between 87 and 97.1%. While the test specificity values ranged from 98.3 to 100% across the platforms for the two serovars (**Table 2**). In all, these data indicate that all platforms show a high degree of accuracy for detecting the two most commonly reported serovars to public health laboratories worldwide (Hendriksen et al., 2011), except for some difficulty with false calls of serovar Typhimurium using the MLST platform, due to the grouping of Typhimurium and its monophasic variant (ssp I 4,[5],12:i:-) into a single category by this method (Achtman et al., 2012). Proper categorization of Enteritidis and Typhimurium is important for some jurisdictions due to strict and costly regulatory controls (Yoshida et al., 2014). While all platforms displayed a high overall success rate the breakdown of the successful results into more informative categories provides a better picture of the results from the platforms.

### Implications of Findings

#### Inconclusive Results

All platforms reported some inconclusive results ranging from a low of 1.1% using SISTR to a high of 30% using SeqSero. Inconclusive results across the platforms were the result of ambiguous calls (multiple serovars listed), lack of serovar variant determination, or a complete lack of serotype reported. Isolates that returned with inconclusive results would require further analysis using the traditional serotyping techniques or other biochemical tests to provide a full serovar call. While these inconclusive results may be of little concern to some users, it may be concerning to others wanting a full answer. For laboratories transitioning away from traditional serotyping that still require a full result this could present a complication, and would require the maintenance of the technical and logistical capacity to carry out traditional serotyping or the submission to a reference laboratory where the generation of results may be delayed. SeqSero specifically displayed difficulty differentiating serovars that have the same antigenic formula but differed on minor O antigenic factors, such as Carrau (6,14,[24]:y:1,7) vs. Madelia (1,6,14,25:y:1,7), or that differed in subspeciation, such as Javiana (subspecies I 1,9,12:l,z28:1,5) vs. subspecies II 9,12:l,z28:1,5. However, there is some evidence that certain minor antigenic factors such as O:6 are variably expressed, which would mean that serovars such as Hadar (6,8:z10:e,n,x) and Istanbul (8:z10:e,n,x) are actually one and the same (Mikoleit et al., 2012). An updated WKL scheme could potentially resolve these issues thereby reducing the number of inconclusive matches recorded by SeqSero; as serovars with these antigenic formulas were reported as both possible options. Meanwhile, the ability of SISTR to resolve ambiguities in the antigenic calls of serovars and the ability of the MLST method to provide a serovar call is only as good as the databases from which they draw their phylogenetic connections to serovars. For rare and unusual serovars this can lead to ambiguous results in SISTR or non-calls by the MLST method and in both these cases traditional serotyping would be required to provide a full serovar call.

#### Incongruent Results

The incongruent isolates uncovered in our validation point toward one of the major implications of using WGS analysis platforms for serotyping, specifically, that a genomic test is not a full equivalent to a phenotypic test in all cases. Using genomics to answer the serovar question requires us to adopt a new paradigm where the carriage of genes for O and H antigens determines the serovar, not the expression of these genes. While this new paradigm presents an opportunity for antigen determination of previously untypeable isolates it also leads to cases of incongruency between *in silico* serotyping tests and traditional methods, specifically in regards to some important monophasic serovars and isolates that are considered rough. For important monophasic serovars such as ssp I 4,[5],12:b:- and ssp I 4,[5],12:i:- previous research has shown that mutations in the flagellar phase variation machinery can lead to the loss of H2 antigen expression without the loss of the H2 antigenic gene determinant, *fljB* (Toboldt et al., 2013; Boland et al., 2015). Multiple mutations in this region have been reported including single base pair or full gene deletions (Toboldt et al., 2013) and insertions of IS26 elements (Boland et al., 2015). Of the 41 monophasic isolates tested in our panel, seven still carried the *fljB* gene and displayed some of the aforementioned mutations within their flagellar phase variation regions (data not shown). Meanwhile rough isolates have lost the ability to express O antigens (Reyes et al., 2012), and evidence suggests that the ‘roughness’ seen in traditional serotyping is not the result of a single genetic loss or change, but instead the result of various mutations, frameshifts or full gene deletions to various genes within the *rfb* region (Kong et al., 2011) which was confirmed through the analysis of our rough isolates.

As we move toward using WGS as a replacement for serotyping it is important that downstream consumers of this information accommodate for the fact that there may be minor changes in the prevalence of key serovars due to isolates possessing antigenic genes that are not expressed. It is important to note that the inconsistencies seen with regards to some monophasic and rough isolates are not the result of incorrect answers, but the result of different ways of framing the serotyping question. Understanding the intricacies of the phenotypic expression of antigenic markers is beyond our current scope of knowledge, and would require detailed studies on the effects of various mutations across a multitude of genes to determine their effect (Ronholm et al., 2016). As surveillance records are important documents for a multitude of users the identification of how the results were generated, version numbers of platforms used, and any limitations of the methods used is crucial so end users know how the results were generated.

#### Incorrect Results

All analysis platforms reported the presence of some incorrect results amongst the 813 isolates tested, ranging from a low of 5.2% of isolates tested using SISTR to just over 11% of isolates tested using SeqSero and MLST. For SeqSero and SISTR some incorrect results were due to incorrect calling of various antigenic determinants, especially in regards to closely related serovars, such as those that differ on the basis of flagellar antigens of the g-complex. This is a limitation of both traditional serotyping (Hendriksen et al., 2009) and some molecular based serotyping techniques (Yoshida et al., 2014) and is related to the high sequence and amino acid similarity amongst some flagellar antigens of the g-complex (McQuiston et al., 2004). As well, novel gene variants were found during the study period including an *fljB* gene for antigen z53 from a subspecies IIIb isolate. While this gene variant was added to the SISTR *fljB* database prior to this study, it points to the fact that these gene databases are only as strong as the data stored in them and highlights the potential for novel gene variants to be discovered in the future. The number of incorrect calls for SISTR and SeqSero was also impacted by an issue where the *rfb* region of the genome was not fully assembled and was split over two contigs. This left the *wzx* and/or *wzy* genes that SISTR uses in its calculation absent from the assembly, leading to a null O-antigen determination, or in the case of the D1 serogroup, a B serogroup prediction. With SISTR, the B serogroup prediction was due to the carriage of the serogroup B *wzy* gene at a separate locus within the genome (Reeves et al., 2013). For SeqSero similarities in the *rfb* region outside the *wzx* lead to incorrect calls (Reeves et al., 2013). The missing sequencing information was attributable to the size selection of the genomic libraries. This region of the genome displays increased genomic fragmentation with the NexteraXT library preparation kit (data not shown), leading to insert sizes well below the 500bp minimum that was selected for. Therefore, this region was frequently filtered out of the genomic library before sequencing, leading to a gap in the sequencing data. Future implementation of *in silico* methods for *Salmonella* serotyping should ensure this size selection step is skipped in the library preparation stage to prevent bias in the sequencing library. Incorrect results from both SISTR and MLST analysis were also attributed to a lack of sufficient representative isolates from a specific serovar/and or phylogenic lineage, throwing off the prediction. MLST analysis calls an isolate based off of the dominant serovar within its database at a specific ST. For example both Typhimurium and ssp I 4,[5],12:i:- isolates are found at ST 19. Since Typhimurium is the dominant serovar at this ST all isolates with this ST are called Typhimurium, leading to an incorrect result for ssp I 4,[5],12:i:- isolates. In SISTR similar results were noted for some ssp I 4,[5],12:b:- isolates who clustered closely with Paratyphi B var. Java representatives. Once again this issue points to the continued need to consistently update and curate the databases used to provide serovar calls.

### Issues to Consider in Transition

While all the tested platforms displayed high levels of successful results, indicating their suitability in maintaining the historical records, surveillance systems, and communication structures on which *Salmonella* serotyping is based, the choice of a single *in silico* serotyping analysis platform for usage by a diagnostic and reference laboratory should be made with consideration for each method’s strengths and weaknesses and the laboratory’s intended purpose. *In silico* serotyping results would be inappropriate for the investigation into the expression of antigenic factors, such as detecting all monophasics or rough isolates, but would be appropriate for routine diagnostic and reference activities. SeqSero provides a genomic understanding of the individual antigens that make up a serovar (Zhang et al., 2015), and represent the closest analogous situation to traditional serotyping. One potential advantage of SeqSero is the possibility of determining the serovar directly from the raw sequencing reads (Zhang et al., 2015). While the raw read analysis through SeqSero was not assessed in this study it may represent a positive for laboratories that lack the computational capacity to assemble genomes prior to serovar determination. MLST analysis uses ST to infer serotypes based on databases that link serovars to specific ST (Achtman et al., 2012), while this method is not analogous to traditional serotyping it allows for enhanced phylogenetic information to be generated that can be used to answer additional epidemiological questions. For example this method allows for the differentiation of *Salmonella* serovar Newport, a polyphyletic serovar, into the three distinct lineages allowing for the potential to further classify polyphyletic serovars (Achtman et al., 2012). Meanwhile SISTR utilizes both methods to provide its overall serovar determination. This not only allows users to gain an understanding of the underlying genetic carriage of the individual antigens, but also allows for the generation of enhanced phylogenetic information that can further classify the isolates (Yoshida et al., 2016b). The combination of both phylogenetic and genomic determinations of a serovar by the SISTR platform allows for significantly stronger result determination as this method led to an overall higher level of successful results as well as a reduction in the number of inconclusive matches that would require further benchtop serotyping. However, all *in silico* serotyping platforms are limited in their ability to define novel serovars, as their databases are only as expansive as what is located within them. Unique ST linkages and sequences of novel gene variants for existing or new antigens must be added to the respective databases before they can be detected and called otherwise they risk displaying an inconclusive or incorrect result.

Ultimately serotyping remains an important tool for public health officials, and the impact of integrating genomic data into existing surveillance systems cannot be ignored. In Canada, serotyping information has formed the basis on which programs such as NESP and PulseNet Canada carry out their mandates (Swaminathan et al., 2006; Government of Canada, 2014). Clinical infections of *Salmonella* are recorded at the serovar level in NESP and are compared to historical levels of disease, allowing for fast and efficient analysis of trends in disease frequencies (Government of Canada, 2014). This information can be used to quickly track *Salmonella* infections, detect outbreaks (Hutwagner et al., 1997), help determine the source of infection (Jackson et al., 2013), and give insight into disease outcomes and potential complications (Gal-Mor et al., 2014). The information provided at this level is then fed into other surveillance programs such as PulseNet Canada, providing the basis on which its databases are organized and also informs the outbreak investigations undertaken (Swaminathan et al., 2006). As Canada’s national diagnostic and reference laboratories with surveillance functions continue to move forward with the implementation of WGS to carry out their mandates, this current paradigm will be shifted and consideration must be given to the impacts this will create. WGS will provide the simultaneous collection of information that will feed into all levels of the current surveillance paradigm, negating the time factor that separates the various programs. *In silico* typing information could be collected for routine identification and diagnostics at the same time whole genome MLST or SNP results are collected for outbreak investigations allowing for the rapid fulfillment of multiple mandates. However, the loss of the time factor that is used in part to separate surveillance programs and their activities should not be used to diminish these programs mandates. Instead the separation between programs will be informed by the resolution of information generated and analyzed. Information collected by NESP on serotype disease frequencies will still be crucial in the allocation of resources spent on investigations by PulseNet Canada. As well, the organization of information within PulseNet Canada’s databases will still rely on serotype information collected by NESP, and the information encoded in serotypes will still be crucial to inform questions during epidemiological investigations into outbreaks.

Further considerations must also be given to the costs associated with sequencing, the technical and informational capacities of national and partner laboratories, turnaround times associated with the batching of isolates, and data sharing models to improve the flow of information between various partners. Following completion of this study, Canada’s reference laboratories are moving forward with the real-time parallel use of SISTR in comparison to traditional serotyping. Roll out of technical and informational capacities is underway within the Canadian system starting with the acquisition of Illumina MiSeq equipment at our partner laboratories and knowledge translation activities between the bioinformatics, microbiological, and epidemiological experts. Utilization of Canada’s bioinformatics infrastructure and data sharing platform, IRIDA, allows stakeholders to maintain local ownership of the sample data while authorizing data sharing to specified partners. Integration of SISTR, as well as the assembly and quality control tools needed to process WGS data, into the IRIDA platform is also underway, creating a one-stop shop of data sources, analysis tools, and investigational activities.

## Conclusion

*Salmonella* serotyping remains an important tool for the public health community and is integral to current public health surveillance systems in Canada. As Canada continues to transition toward using WGS to carry out its public health mandate, backward compatibility with existing surveillance systems is important. Three *in silico* serotyping platforms were validated as part of this study, SISTR, SeqSero, and MLST analysis, and we reported successful results in 94.8, 88.2, and 88.3% of the 813 isolates tested, with SISTR significantly outperforming both SeqSero and MLST. However, all platforms would be suitable for maintaining the historical records, surveillance systems, and communication structures currently in place. Results also point to the need to reframe our understanding of serotyping within the genomic era. Use of SISTR or SeqSero provides us with an understanding of the antigenic genes that are carried by an isolate and not necessarily what is expressed by that isolate. While this may lead to incongruences between the two methods, it is important to note these incongruences are not the result of errors but just a conceptual shift in how we will be defining what a serovar is. Both SISTR and MLST also provide us with increased phylogenetic classification which can be used to answer additional epidemiological questions; while SeqSero provides the opportunity to analyze results directly from raw reads. Ultimately the choice of system will depend on users need. The adoption of WGS by diagnostic and reference laboratories with surveillance functions will provide the simultaneous collection of information that will feed into multiple levels of the current surveillance paradigm, but does not negate the importance of the various programs and the role of serotyping in these programs.

## Author Contributions

Conceived and designed the experiment: CAY, CY, JN, ET, AR, AN, CN, and PNSC. Performed the experiments: CAY, JR, PK, and MW. Contributed analysis: CAY, CY, PK, MW, SC, and CN. Wrote the paper: CAY and CY.

## Conflict of Interest Statement

CY, JN, PK, and ET were part of the SISTR development team. The other authors declare that the research was conducted in the absence of any commercial or financial relationships that could be construed as a potential conflict of interest.

## Footnotes

1 http://www.denglab.info/SeqSero

2 https://lfz.corefacility.ca/sistr-app/

3 http://ccb.jhu.edu/software/FLASH

4 http://bioinf.spbau.ru/spades

5 https://lfz.corefacility.ca/sistr-app/

6 http://www.denglab.info/SeqSero

7 http://mlst.warwick.ac.uk/mlst/

8 http://www.graphpad.com/quickcalcs
