# Supplementary material for: The Validation and Implications of Using Whole Genome Sequencing as a Replacement for Traditional Serotyping for a National Salmonella Reference Laboratory
Source: Front Microbiol. 2017 Jun 9;8:1044. doi: 10.3389/fmicb.2017.01044 (PMC5465390; doi:10.3389/fmicb.2017.01044)
Supplement: Supplementary file 1 [file Table_1.DOCX]

Supplementary Material

The Validation and Implications of Using Whole Genome Sequencing as a Replacement for Traditional Serotyping for a National *Salmonella* Reference Laboratory

Chris A. Yachison, Catherine Yoshida, James Robertson, John H. E. Nash, Peter Kruczkiewicz, Eduardo N. Taboada, Matthew Walker, Aleisha Reimer, Sara Christianson, Anil Nichani, the PulseNet Canada Steering Committee and Celine Nadon*

*** Correspondence:** Celine Nadon: celine.nadon@phac-aspc.gc.ca

# Supplementary Table

Supplementary Table 1: Performance of the three *in silico* methods for *Salmonella* serovar prediction, SISTR, SeqSero, and MLST compared to traditional serotyping for 813 Canadian clinical isolates from 38 serovar categories.

| **Serovar Groups** | **Total Tested** | **Full** | | | **Inconclusive** | | | **Incongruent** | | | **Incorrect** | | |
| --- | --- | --- | --- | --- | --- | --- | --- | --- | --- | --- | --- | --- | --- |
|  |  | **SISTR** | **SeqSero** | **MLST** | **SISTR** | **SeqSero** | **MLST** | **SISTR** | **SeqSero** | **MLST** | **SISTR** | **SeqSero** | **MLST** |
| **Agona** | **23** | 22 | 22 | 22 | 0 | 0 | 1 | 0 | 0 | 0 | 1 | 1 | 0 |
| **Braenderup** | **23** | 23 | 17 | 23 | 0 | 0 | 0 | 0 | 0 | 0 | 0 | 6 | 0 |
| **Carrau** | **5** | 5 | 0 | 0 | 0 | 5 | 0 | 0 | 0 | 0 | 0 | 0 | 5 |
| **Cerro** | **8** | 8 | 0 | 7 | 0 | 5 | 0 | 0 | 0 | 0 | 0 | 3 | 1 |
| **Corvallis** | **8** | 8 | 0 | 8 | 0 | 8 | 0 | 0 | 0 | 0 | 0 | 0 | 0 |
| **Dublin** | **7** | 7 | 4 | 4 | 0 | 0 | 0 | 0 | 0 | 0 | 0 | 3 | 3 |
| **Enteritidis** | **42** | 40 | 34 | 40 | 1 | 2 | 1 | 0 | 0 | 0 | 1 | 6 | 1 |
| **Hadar** | **27** | 26 | 0 | 26 | 0 | 26 | 0 | 0 | 0 | 0 | 1 | 1 | 1 |
| **Heidelberg** | **28** | 28 | 27 | 28 | 0 | 1 | 0 | 0 | 0 | 0 | 0 | 0 | 0 |
| **I 4,[5],12:b:-** | **20** | 11 | 0 | 11 | 0 | 14 | 0 | 6 | 6 | 6 | 3 | 0 | 3 |
| **I 4,[5],12:i:-** | **21** | 18 | 0 | 0 | 0 | 20 | 0 | 1 | 1 | 1 | 2 | 0 | 20 |
| **Infantis** | **27** | 27 | 23 | 27 | 0 | 0 | 0 | 0 | 0 | 0 | 0 | 4 | 0 |
| **Javiana** | **23** | 23 | 0 | 23 | 0 | 20 | 0 | 0 | 0 | 0 | 0 | 3 | 0 |
| **Kouka** | **1** | 1 | 0 | 0 | 0 | 0 | 1 | 0 | 0 | 0 | 0 | 1 | 0 |
| **Lattenkamp** | **2** | 2 | 2 | 0 | 0 | 0 | 2 | 0 | 0 | 0 | 0 | 0 | 0 |
| **Madelia** | **2** | 2 | 0 | 0 | 0 | 2 | 0 | 0 | 0 | 0 | 0 | 0 | 2 |
| **Montevideo** | **27** | 26 | 21 | 25 | 0 | 0 | 1 | 0 | 0 | 0 | 1 | 6 | 1 |
| **Muenchen** | **23** | 23 | 0 | 23 | 0 | 23 | 0 | 0 | 0 | 0 | 0 | 0 | 0 |
| **Newport** | **27** | 26 | 26 | 26 | 0 | 0 | 0 | 0 | 0 | 0 | 1 | 1 | 1 |
| **Oranienburg** | **23** | 22 | 0 | 20 | 0 | 18 | 3 | 0 | 0 | 0 | 1 | 5 | 0 |
| **Panama** | **8** | 7 | 0 | 8 | 0 | 7 | 0 | 0 | 0 | 0 | 1 | 1 | 0 |
| **Paratyphi A** | **23** | 23 | 22 | 23 | 0 | 0 | 0 | 0 | 0 | 0 | 0 | 1 | 0 |
| **Paratyphi B** | **5** | 5 | 0 | 5 | 0 | 5 | 0 | 0 | 0 | 0 | 0 | 0 | 0 |
| **Paratyphi B var. Java** | **26** | 24 | 0 | 6 | 0 | 25 | 0 | 0 | 0 | 0 | 2 | 1 | 20 |
| **Rough O non ssp I** | **5** | 0 | 0 | 0 | 0 | 0 | 0 | 5 | 5 | 5 | 0 | 0 | 0 |
| **Rough O ssp I** | **21** | 0 | 0 | 0 | 0 | 0 | 0 | 21 | 21 | 21 | 0 | 0 | 0 |
| **Saintpaul** | **23** | 23 | 23 | 21 | 0 | 0 | 2 | 0 | 0 | 0 | 0 | 0 | 0 |
| **Sandiego** | **8** | 8 | 8 | 0 | 0 | 0 | 0 | 0 | 0 | 0 | 0 | 0 | 8 |
| **Schwarzengrund** | **8** | 8 | 8 | 8 | 0 | 0 | 0 | 0 | 0 | 0 | 0 | 0 | 0 |
| **Senftenberg** | **7** | 7 | 0 | 7 | 0 | 6 | 0 | 0 | 0 | 0 | 0 | 1 | 0 |
| **ssp II** | **8** | 6 | 1 | 0 | 0 | 4 | 8 | 0 | 0 | 0 | 2 | 3 | 0 |
| **ssp IIIa** | **8** | 7 | 3 | 1 | 0 | 4 | 7 | 0 | 0 | 0 | 1 | 1 | 0 |
| **ssp IIIb** | **11** | 9 | 2 | 0 | 0 | 5 | 11 | 0 | 0 | 0 | 2 | 4 | 0 |
| **ssp IV** | **7** | 7 | 2 | 0 | 0 | 5 | 1 | 0 | 0 | 0 | 0 | 0 | 6 |
| **Stanley** | **24** | 24 | 24 | 24 | 0 | 0 | 0 | 0 | 0 | 0 | 0 | 0 | 0 |
| **Thompson** | **23** | 23 | 20 | 23 | 0 | 1 | 0 | 0 | 0 | 0 | 0 | 2 | 0 |
| **Typhi** | **23** | 22 | 18 | 23 | 0 | 0 | 0 | 0 | 0 | 0 | 1 | 5 | 0 |
| **Typhimurium** | **39** | 39 | 38 | 39 | 0 | 0 | 0 | 0 | 0 | 0 | 0 | 1 | 0 |
| **Non-Target** | **169** | 139 | 95 | 132 | 8 | 38 | 14 | 0 | 0 | 0 | 22 | 36 | 23 |
| **Totals** | **813** | **729** | **440** | **633** | **9** | **244** | **52** | **33** | **33** | **33** | **42** | **96** | **95** |
